# Dietary intake of non-dialysis chronic kidney disease patients: the PROGREDIR study. A cross-sectional study

**DOI:** 10.1590/1516-3180.2017.0177141217

**Published:** 2018-06-18

**Authors:** Alisson Diego Machado, Fernanda Silva Nogueira dos Anjos, Maria Alice Muniz Domingos, Maria del Carmen Bisi Molina, Dirce Maria Lobo Marchioni, Isabela Judith Martins Benseñor, Silvia Maria de Oliveira Titan

**Affiliations:** I MSc. Dietitian, Department of Nephrology, Hospital das Clínicas (HC), Faculdade de Medicina da Universidade de São Paulo (FMUSP), São Paulo (SP), Brazil.; II Dietitian, Department of Nephrology, Hospital das Clínicas (HC), Faculdade de Medicina da Universidade de São Paulo (FMUSP), São Paulo (SP), Brazil.; III MD. Nephrologist, Department of Nephrology, Hospital das Clínicas (HC), Faculdade de Medicina da Universidade de São Paulo (FMUSP), São Paulo (SP), Brazil.; IV PhD. Dietitian and Associate Professor, Health Sciences Center, Universidade Federal do Espírito Santo (UFES), Vitória (ES), Brazil.; V PhD. Dietitian and Associate Professor, Department of Nutrition, Faculdade de Saúde Pública (FSP), Universidade de São Paulo (USP), São Paulo (SP), Brazil.; VI MD. Associate Professor, General Medicine Unit, Hospital Universitário (HU), Faculdade de Medicina da Universidade de São Paulo (FMUSP), São Paulo (SP), Brazil.; VII MD. Research Investigator, Department of Nephrology, Hospital das Clínicas (HC), Faculdade de Medicina da Universidade de São Paulo (FMUSP), São Paulo (SP), Brazil.

**Keywords:** Renal insufficiency, chronic, Diet, Health surveys

## Abstract

**BACKGROUND:**

Despite evidence that diet is very important in relation to chronic kidney disease (CKD) progression, studies in this field are scarce and have focused only on some specific nutrients. We evaluated the energy, macronutrient and micronutrient intakes and dietary patterns of non-dialysis CKD participants in the PROGREDIR study.

**DESIGN AND SETTING:**

Cross-sectional study; CKD cohort, São Paulo, Brazil.

**METHODS:**

Baseline data on 454 participants in the PROGREDIR study were analyzed. Dietary intake was evaluated through a food frequency questionnaire. Dietary patterns were derived through principal component analysis. Energy and protein intakes were compared with National Kidney Foundation recommendations. Linear regression analysis was performed between energy and nutrient intakes and estimated glomerular filtration rate (eGFR), and between sociodemographic and clinical variables and dietary patterns.

**RESULTS:**

Median energy and protein intakes were 25.0 kcal/kg and 1.1 g/kg, respectively. In linear regression, protein intake (β = -3.67; P = 0.07) was related to eGFR. Three dietary patterns (snack, mixed and traditional) were retained. The snack pattern was directly associated with male gender (β = 0.27; P = 0.006) and inversely with diabetes (β = -0.23; P = 0.02). The traditional pattern was directly associated with male gender (β = 0.27; P = 0.007) and schooling (β = 0.40; P < 0.001) and inversely with age (β = -0.01; P = 0.001) and hypertension (β = -0.34; P = 0.05).

**CONCLUSIONS:**

We identified low energy and high protein intake in this population. Protein intake was inversely related to eGFR. Dietary patterns were associated with age, gender, schooling level, hypertension and diabetes.

## INTRODUCTION

Chronic kidney disease (CKD), defined as estimated glomerular filtration rate (eGFR) < 60 ml/min/1.73m^2^ or persistent albuminuria, affects more than 10% of the world’s population.^1^^,^^2^ Early diagnosis of CKD is important for reducing the risk of progression and cardiovascular morbidity and mortality, which is 30 times greater among people with CKD than in the general population.^3^ Management of modifiable risk factors is essential, and diet has emerged as an important but often neglected therapeutic tool for prevention and retardation of CKD progression.^4^

Currently, the main goals of nutritional therapy in relation to CKD are to reduce accumulation of byproducts from metabolism and reduce progression of renal disease.^5^ In addition, diet may form a strategy for preventing or ameliorating complications of CKD, including acidosis, hyperkalemia, hyperphosphatemia, uremic symptoms, bone diseases and protein-energy wasting.^6^^,^^7^ However, diet is still underused as a prevention strategy.^8^ Furthermore, although low-protein diets are the strategies that have been most studied in relation to CKD, there is evidence to suggest that many other nutrients may influence renal outcomes,^4^ such as phosphorus, sodium, potassium, calcium and vitamin K. In addition, few studies have addressed new analytical approaches, such as dietary pattern analysis, rather than evaluation of individual nutrients.^7^

PROGREDIR is a cohort study that was designed to evaluate the determinants of CKD progression and mortality risk among CKD patients.^9^ The cohort essentially comprises people with CKD classes 3 and 4 living in São Paulo, Brazil, and diet is one of the factors under investigation.

## OBJECTIVE

We evaluated the association between energy, macronutrient and micronutrient intakes and eGFR, along with dietary patterns and their associated factors among the participants of the PROGREDIR study.

## METHODS

The present study consisted of an evaluation on baseline data from the PROGREDIR study. Details of the methods have been published elsewhere.^9^ Briefly, patients attending the outpatient service of Hospital das Clínicas, São Paulo, a quaternary-level care facility, were invited to participate in the study. Initially, from the outpatient records, all patients aged ≥ 30 years old who presented at least two creatinine measurements (with a minimum interval of three months) ≥ 1.6 mg/dl for men and ≥ 1.4 mg/dl for women were considered to be potential candidates. Patients who were attending oncology, psychiatry, urology, human immunodeficiency virus/acquired immunodeficiency syndrome (HIV/AIDS), viral hepatitis and glomerulonephritis services were excluded. The remaining candidates were then contacted by phone and invited to participate if none of the following exclusion criteria were met: hospitalization or acute myocardial infarction in the last six months, autoimmune diseases, pregnancy, psychiatric diseases, ongoing chemo or immunosuppressive therapy, ongoing renal replacement therapy, glomerulonephritis, HIV/AIDS infection, hepatitis B or C, or previous transplantation of any organ. Recruitment took place from March 2012 to December 2013, and 454 participants were enrolled. The study was approved by two local ethics committees, and written informed consent was obtained from all participants (protocol number 11147/11, approved on November 4, 2011, and protocol number 0798/11, approved on February 2, 2012).

Each participant visited the research center for interviews and clinical examinations in accordance with standard protocols. The interviews and clinical examinations were conducted by trained personal under strict quality control conditions. Data on sociodemographic variables (age, gender, schooling level and income class) and lifestyle variables (tobacco use, alcohol use and physical activity practice) were self-reported. Diabetes was defined using a five-criterion definition that included any previous medical history of diabetes, use of medication to treat diabetes, fasting plasma glucose ≥ 126 mg/dl, glycated hemoglobin ≥ 6.5%, and two-hour plasma glucose ≥ 200 mg/dl (oral glucose tolerance test). eGFR was estimated by means of the Chronic Kidney Disease Epidemiology Collaboration equation.^10^

We used the validated food frequency questionnaire (FFQ) of the Brazilian Longitudinal Study of Adult Health (ELSA-Brasil)^11^ to evaluate the dietary intake. The questionnaire asked about 114 foods or preparations and evaluated the frequency (daily, weekly or monthly) and the usual amount of intake of each food/preparation (in household measurements). In addition, it also included 19 questions about the characteristics of the subjects’ dietary habits over the last 12 months. This FFQ was applied by staff who had been trained for this function. After data collection, the FFQ was reviewed to verify whether the portion size of the foods was in accordance with what is usually consumed by the Brazilian population.

To evaluate energy and nutrient intakes, we used the United States Department of Agriculture (USDA) Food Composition Databases^12^ except when these values were outside of the range of 80% to 120% of the values in the Brazilian Table of Food Composition,^13^ in which case we used the latter values. We excluded patients whose energy intake was higher than 5,000 kcal (n = 11) from the analyses, because these are unlikely values that might have led to overestimation of nutrient intakes.^14^

Macronutrient and micronutrient intakes were adjusted for energy using the residual method.^15^ To analyze energy and protein intake per kg, we used the current body weight, or an adjusted weight when body mass index adequacy was less than 95% or greater than 115%.^16^ The energy and protein intakes were compared with the National Kidney Foundation recommendations.^16^ Intakes of supplements and medications were not taken into consideration in the current analyses.

Dietary patterns were derived from principal component analysis, with orthogonal (varimax) rotation to extract factors. We considered the daily frequency of intake of each food in the analyses. Subsequently, foods with similar nutritional compositions were grouped into 20 foods/food groups. An exploratory factor analysis was performed, and the adequacy of the data was evaluated by means of the Kaiser-Meyer-Olkin (KMO) test and the Bartlett test of sphericity (BTS). We set different numbers of factors and chose those with interpretable patterns, which were named according to the interpretation of the data. A score was determined for each pattern, which allowed each participant to have one factor score for all patterns identified.^17^

Energy and nutrient intakes were presented as means and standard deviations or as medians and interquartile ranges, according to gender. The variables were tested for normal distribution using the Kolmogorov-Smirnov test, and then differences between the groups were tested using Student’s t test (normal distribution) or the Mann-Whitney test (non-normal distribution). Linear regression analysis was performed between energy and nutrient intakes and eGFR as a dependent variable and between sociodemographic and clinical variables and dietary patterns (factor scores) as a dependent variable. All analyses were performed using the SPSS software, version 17.0.

## RESULTS

The baseline characteristics of the participants included in the study are described in Table 1. There was a predominance of elderly, male, hypertensive and diabetic participants. The mean eGFR was 38.4 ± 14.6 (ml/min/1.73 m^2^).

**Table 1:** Baseline characteristics of participants in the PROGREDIR study

Variable*	All n = 454	Male n = 287	Female n = 167	P^†^
Sociodemographic variables				
Age, years	68 (60-76)	68 (61-76)	69 (59-77)	0.49
Schooling (≤ 8 years of study), n (%)	287 (63.2)	167 (58.2)	120 (71.9)	0.004
Lower middle class, n (%)	248 (54.6)	142 (49.5)	106 (63.5)	0.004
Lifestyle variables				
Tobacco use, n (%)	41 (9.0)	27 (9.5)	14 (8.4)	< 0.001
Alcohol use, n (%)	171 (37.7)	132 (46.2)	39 (23.4)	< 0.001
Physical activity practice, n (%)	137 (30.2)	104 (36.9)	33 (20.0)	< 0.001
Clinical and laboratory variables				
Hypertension, n (%)	416 (91.6)	262 (91.3)	154 (92.2)	0.73
Diabetes, n (%)	257 (56.6)	167 (58.2)	90 (53.9)	0.37
eGFR, ml/min/1.73 m^2^	38.4 ± 14.6	40.4 ± 15.4	34.8 ± 12.4	< 0.001
Serum urea, mg/dl	69 (54-89)	69 (54-87)	70 (55-93)	0.33
Microalbuminuria, mg/g creatinine	83 (15-668)	70 (14-619)	94 (21-813)	0.32
Serum phosphorus, mg/dl	3.6 ± 0.6	3.6 ± 0.6	3.8 ± 0.6	< 0.001
Serum potassium, mEq/l	4.6 ± 0.5	4.6 ± 0.5	4.5 ± 0.5	0.25
Glycated hemoglobin, %	6.2 (5.8-7.2)	6.2 (5.7-7.2)	6.2 (5.9-7.2)	0.59
Total cholesterol, mg/dl	166 (140-191)	157 (133-180)	179 (159-209)	< 0.001
LDL-C, mg/dl	88 (68-109)	81 (63-105)	94 (75-124)	< 0.001
HDL-C, mg/dl	44 (37-53)	41 (34-48)	49 (42-58)	< 0.001
Triglycerides, mg/dl	142 (99-193)	142 (99-188)	140 (97-202)	0.57
Systolic blood pressure, mmHg	140 ± 24	139 ± 24	142 ± 25	0.19
Diastolic blood pressure, mmHg	75 (67-84)	75 (67-85)	75 (67-82)	0.55
Anthropometric measurements				
Body mass index, kg/m^2^	29 (26-32)	29 (26-32)	29 (25-33)	0.66
Body fat, %	30 (27-34)	28 (26-31)	35 (32-40)	< 0.001

^*^Continuous variables: mean ± standard deviation or median (with interquartile range); categorical variables: number (with percentage); ^†^P-value for comparison between gender groups. eGFR = estimated glomerular filtration rate; HDL-C = high-density lipoprotein; LDL-C = low-density lipoprotein.

Regarding energy and nutrient intakes, 293 (66.1%) of the participants showed an energy intake below the recommended amount, while 399 (90.1%) of them had a protein intake above the recommended amount for non-dialysis CKD patients. The male patients presented statistically higher intakes of energy (kcal) and iron than those of the females, who presented higher intake of protein (g/kg), dietary fiber, vitamin A, vitamin E, thiamine, pantothenic acid, cobalamin, vitamin C and potassium (Table 2).

**Table 2:** Energy and nutrient intakes among all participants in the PROGREDIR study and according to gender

Energy/Nutrient^*^	Intake^†^			P^‡^
	All n = 443	Male n = 277	Female n = 166	
Energy, kcal	1923 (1491-2489)	2105 (1611-2684)	1625 (1286-2152)	< 0.001
Energy, kcal/kg	25.0 (19.5-33.0)	26.3 (19.9-34.0)	24.3 (19.1-31.6)	0.10
Protein, g	83 (72-97)	83 (73-96)	83 (72-98)	0.73
Protein, g/kg	1.1 (0.9-1.4)	1.0 (0.9-1.2)	1.3 (1.0-1.5)	< 0.001
Carbohydrate, g	289 ± 41	288 ± 44	290 ± 37	0.49
Total fat, g	50 ± 11	51 ± 11	49 ± 10	0.14
Dietary fiber, g	26.2 ± 8.5	25.4 ± 8.3	27.5 ± 8.6	0.01
Vitamin A, µg RAE	328 (236-505)	321 (231-480)	354 (253-587)	0.02
Vitamin E, mg	6.4 (5.2-7.9)	6.1 (5.0-7.5)	6.9 (5.7-8.5)	< 0.001
Vitamin K, µg	160 (106-249)	160 (102-246)	160 (108-260)	0.44
Thiamine, mg	1.3 (1.0-1.8)	1.2 (1.0-1.6)	1.4 (1.0-2.1)	0.001
Riboflavin, mg	1.3 (0.9-1.8)	1.3 (0.9-1.8)	1.4 (0.9-1.8)	0.77
Niacin, mg	20.8 (15.0-31.1)	21.1 (15.4-30.4)	20.5 (14.7-34.1)	0.86
Pyridoxine, mg	0.7 (0.5-0.9)	0.7 (0.5-0.9)	0.7 (0.5-0.9)	0.34
Folate, µg	520 (447-608)	523 (439-611)	513 (453-603)	0.95
Cobalamin, µg	3.7 (2.8-4.8)	3.6 (2.7-4.7)	4.1 (3.0-5.3)	0.004
Vitamin C, mg	151 (74-261)	135 (65-221)	193 (104-311)	< 0.001
Magnesium, mg	276 (240-329)	274 (240-318)	284 (242-342)	0.15
Manganese, mg	2.9 (2.4-3.5)	2.8 (2.4-3.4)	3.0 (2.4-3.6)	0.24
Calcium, mg	737 (539-974)	714 (533-959)	787 (549-990)	0.11
Iron, mg	10.2 ± 2.4	10.4 ± 2.4	9.9 ± 2.4	0.04
Zinc, mg	9.7 (8.4-11.8)	9.9 (8.6-11.8)	9.5 (8.0-11.8)	0.09
Selenium, µg	120 (104-139)	119 (102-136)	120 (106-142)	0.24
Phosphorus, mg	1184 ± 232	1178 ± 223	1196 ± 247	0.43
Sodium, mg	2236 (1868-2547)	2217 (1866-2613)	2241 (1875-2468)	0.57
Potassium, mg	3044 ± 700	2985 ± 681	3143 ± 720	0.02

^*^Nutrient intakes after adjustment for energy, by means of residual method; ^†^mean ± standard deviation or median (with interquartile range); ^‡^P-value for comparison between gender groups. RAE = retinol activity equivalent.

In the univariate linear regression analysis, protein intake (g/kg) was inversely related to eGFR, while pyridoxine intake was directly associated. After adjustment for age, gender, diabetes, microalbuminuria and systolic blood pressure, only protein intake (g/kg) showed a trend towards remaining inversely related to eGFR (Table 3).

**Table 3:** Linear regression between nutrient intakes and eGFR among participants in the PROGREDIR study

Variable	β	95% CI	P
Model 1 - Univariate regression			
Protein, g/kg	-6.26	-10.10- -2.41	0.001
Pyridoxine, mg	4.52	0.18-8.86	0.04
Model 2 - Variables adjusted for age, gender, diabetes, microalbuminuria and systolic blood pressure			
Protein, g/kg	-3.67	-7.60-0.26	0.07
Pyridoxine, mg	2.68	-1.53-6.88	0.21

Dependent variable: eGFR = estimated glomerular filtration rate; CI = confidence interval.

Three dietary patterns were retained for subsequent analysis. The snack pattern was composed predominantly of breads, biscuits, cakes, farinaceous products, butter, margarine, eggs, processed meat, sweets, snacks, whole dairy products and sweetened beverages, which explained 12.6% of the variance. The mixed pattern was composed of whole grains, pasta, tubers, red meat, poultry, fish, seafood, fruits, vegetables, low-fat dairy products and natural juice, which explained 8.9% of the variance. The traditional pattern was composed of white rice, beans and coffee, which explained 7.0% of the variance. These patterns are shown in Table 4. The value from the KMO test was 0.601 and the P-value of the BTS was < 0.001.

**Table 4:** Distribution of factor loadings of dietary patterns identified among participants in the PROGREDIR study

Food or food group	Dietary pattern		
	Snack	Mixed	Traditional
White rice	-0.058	0.232	0.809
Breads, biscuits, cakes and farinaceous products	0.586	0.099	0.154
Whole grains	-0.146	0.300	-0.597
Pasta and tubers	0.146	0.358	0.144
Butter and margarine	0.521	0.133	0.027
Eggs	0.341	0.308	0.115
Red meat	0.190	0.315	0.087
Processed meat (sausages, hamburgers, ham, mortadella, bacon, canned sardines)	0.558	0.160	0.037
Poultry	0.105	0.427	0.000
Fish and seafood	-0.029	0.440	-0.030
Beans (beans, *feijoada*, lentil, chickpeas, peas)	-0.003	0.276	0.613
Fruits	0.178	0.552	-0.196
Vegetables	-0.047	0.658	0.101
Sweets (ice cream, candies, gelatin, chocolate, pudding, fruit jam, honey)	0.437	0.106	-0.155
Snacks (*pão de queijo*, pizza, *esfiha*, *pastel*, *coxinha*, hot dog, popcorn)	0.237	0.123	0.028
Coffee	0.245	-0.005	0.277
Whole dairy products	0.609	-0.086	0.023
Low-fat dairy products	-0.222	0.374	-0.375
Natural juice	0.078	0.323	0.029
Sweetened beverages (soda, fruit nectar)	0.504	-0.162	0.238
% of variance explained	12.6	8.9	7.0
Cumulative % of variance	12.6	21.5	28.5

Values in bold indicate greater adherence of a food or food group to the dietary pattern.

The snack pattern was directly associated with male gender and inversely related to diabetes. The traditional pattern was directly associated with male gender and schooling level and inversely related to age and hypertension (Table 5). None of the patterns was significantly related to eGFR.

**Table 5:** Linear regression between sociodemographic and clinical variables and the dietary patterns among participants in the PROGREDIR study

Variable	Dietary pattern					
	Snack		Mixed		Traditional	
	β	P	β	P	β	P
Model 1 - Univariate regression						
Age, years	0.002	0.55	0.002	0.60	-0.01	0.001
Male gender	0.27	0.006	-0.05	0.61	0.27	0.007
Schooling level (≤ 8 years of study)	0.01	0.91	-0.11	0.25	0.23	0.02
Hypertension	0.08	0.66	-0.13	0.45	-0.40	0.03
Diabetes	-0.22	0.03	0.17	0.08	-0.12	0.20
eGFR, ml/min/1.73 m^2^	0.003	0.29	0.001	0.73	0.006	0.08
BMI, kg/m^2^	-0.01	0.28	-0.02	0.06	0.002	0.86
Model 2 - Variables adjusted for age and gender						
Schooling level (≤ 8 years of study)	0.02	0.83	-0.16	0.11	0.40	< 0.001
Hypertension	0.06	0.76	-0.16	0.38	-0.34	0.05
Diabetes	-0.23	0.02	0.15	0.11	-0.13	0.18
eGFR, ml/min/1.73 m^2^	0.002	0.49	0.002	0.62	0.002	0.47
BMI, kg/m^2^	-0.007	0.47	-0.02	0.09	-0.002	0.81

Dependent variables: factor scores for each dietary pattern. BMI = body mass index; eGFR = estimated glomerular filtration rate.

## DISCUSSION

In the present study, the participants reported having low energy and high protein intakes in relation to the nutritional recommendations for CKD patients. This is noteworthy, considering that this population was recruited from outpatient services in one of the major public hospitals in São Paulo. This finding is concordant with data from other studies that have also reported low energy and high protein intake among non-dialysis CKD patients, and it highlights the difficulty in achieving efficacious application of nutritional guidelines in cases of chronic diseases. In a study by Avesani et al.,^18^ an energy intake of 22.4 kcal/kg was identified among Brazilian patients, which was lower than what was observed in the present study. The energy and protein intake of the sample of the present study was similar to that found in the Modification of Diet in Renal Disease (MDRD) study, in an American population with CKD, but was higher than what was found in the Chronic Renal Insufficiency Cohort (CRIC) study and the National Health and Nutrition Examination Survey (NHANES III) study.^19^

Although a low-protein diet is currently the main therapeutic dietary recommendation for CKD, its actual application in clinical settings varies widely. Several factors may be contributing towards this, such as difficulty in establishing multidisciplinary approaches, lack of adoption of low-protein diets, fear of intensification of protein-energy wasting and low adherence to treatment by patients.^20^ Nonetheless, it was surprising to observe that 90% of the CKD population in the PROGREDIR study reported having a protein intake above the recommended value. There may have been several reasons for this, and these were not evaluated in the present study, but they possibly include the dietary habits of the Brazilian population, which are known to include high animal protein intake,^21^ along with low adherence to treatment and lack of use of dietary interventions as an important tool for medical treatment. These results show that implementation of low-protein diets is not being accomplished in this CKD population, despite the fact that these individuals mostly presented CKD of classes 3 and 4 and were at high risk of CKD progression.

In addition, in the present study, protein intake showed a strong trend towards being inversely associated with eGFR in the linear regression analysis. Although we cannot address causality in this cross-sectional study, this finding is concordant with data from other studies that have suggested that protein intake is associated with CKD progression. These studies form the basis for the dietary recommendation of lowering protein intake to less than 0.8 g/kg/day.^16^

The low energy intake in this population may have been due to several factors, such as anorexia, nausea, anemia, restrictive diets and comorbidities. Low energy intake is one of the factors associated with the development of protein-energy wasting,^22^ which is related to increased morbidity and mortality in CKD cases.^23^ Although it has been shown that low energy intake is related to lower eGFR and higher serum creatinine and blood urea nitrogen,^24^ our study did not show any significant relationship between eGFR and energy intake.

There is a lack of Brazilian studies evaluating the micronutrient intakes of non-dialysis CKD patients. In a study that evaluated the zinc and iron content in the diets of Brazilian non-dialysis CKD patients, low content of these minerals in comparison with those of the present study were observed.^25^ We were unable to identify any other Brazilian studies evaluating the micronutrient intakes of non-dialysis CKD patients.

In a study conducted in Poland that evaluated the dietary intake of non-dialysis female CKD patients by means of a three-day food record, the intake was lower than in the present study for all micronutrients except for vitamin A, vitamin E and pyridoxine.^26^ In three large American cohort studies (MDRD, CRIC and NHANES III), high phosphorus intake was observed,^19^ as in the present study, in which an amount of 1184 mg was recorded, i.e. almost 60% above the recommended amount.^27^ However, in the present study, the sodium intake was lower than in those studies and the potassium intake was slightly higher.^19^

The dietary patterns identified in the present study were similar to those of other studies conducted among with Brazilian adults and elderly people with normal eGFR.^28^^,^^29^^,^^30^ According to the value found in the KMO test and the P-value of the BTS, the factor analysis can be considered adequate.^31^ In addition, the accumulated variance was similar to that of other studies.^28^^,^^32^

We emphasize that the mixed pattern, composed of whole grains, meats, fruits and vegetables is generally referred to as “healthy” or “prudent” in studies on dietary patterns.^28^^,^^30^^,^^33^ However, because a low-protein diet is recommended for non-dialysis CKD patients, we consider that high intake of meats, and consequently protein, may not be healthy or prudent in this population, and thus we name this pattern “mixed.”

The traditional pattern was directly associated with male gender and inversely associated with age, as found by Cardoso et al.^34^ in a population study. Male gender was also associated with the snack pattern, as verified by Ferreira et al.,^32^ which may indicate a minor concern regarding feeding among men. The snack pattern was inversely associated with presence of diabetes, which may suggest that the diabetic subjects altered their dietary pattern because of their disease, which may explain why the diabetics consumed higher amounts of protein, vitamins and minerals than did the non-diabetic participants (data not shown).

In our study, none of the dietary patterns were associated with eGFR. In accordance with this finding, Gutiérrez et al.^35^ showed in a cohort study that no dietary pattern was related to CKD progression. However, a diet rich in fruits and vegetables was associated with lower risk of mortality. In addition, in a recent meta-analysis, there was no association between a healthy pattern (higher in fruits, vegetables, fish, cereals and whole grains and lower in red meat and refined sugars) and the risk of end-stage renal disease, but it was found that this pattern was associated with lower risk of mortality.^36^

In studies that included participants without baseline kidney disease, the results have been different. In a subgroup analysis from the Nurses’ Health Study, the Western pattern (rich in red and processed meats, saturated fats and sweets) was directly related to decreased eGFR and microalbuminuria, while the DASH (Dietary Approaches to Stop Hypertension) pattern was inversely associated with decreased eGFR.^37^ In the ULSAM (Uppsala Longitudinal Study of Adult Men) cohort, higher adherence to the Mediterranean diet was associated with lower presence of CKD and higher survival rates.^38^

These results may indicate that dietary patterns have less influence on the risk of end-stage renal disease after CKD has already become established and has reached moderate to advanced stages. However, higher intake of fruits and vegetables appears to be beneficial in relation to the risk of mortality among people with impaired and normal kidney function. Further studies may confirm these findings and also evaluate the association between dietary patterns and other factors, such as cardiovascular risk.

Our study had some limitations. Firstly, it was a cross-sectional analysis. Secondly, the PROGREDIR population is a hospital-derived sample, which implies that the diet reported was possibly influenced by current illnesses and their treatments. This may have reduced the extent to which the dietary assessment reflected the long-term previous intake. Furthermore, FFQs are limited instruments that may not include all foods consumed, which therefore may impair quantification of nutrient intakes. The participants who could have underreported their energy intake were not excluded from the analyses. However, we used the residual method to adjust the intake of all nutrients by energy, obtaining the intake data without the influence of energy.^15^ Despite these limitations, the study included a representative sample and used an appropriate method of analysis. Hence, it showed interesting results regarding the dietary profile and patterns of a CKD population.

## CONCLUSION

We found low energy intake and high protein intake in a CKD population, thus demonstrating the need for nutritional intervention. In addition, protein intake was inversely related to eGFR. Dietary patterns were not associated with eGFR, but were associated with age, gender, schooling level and presence of hypertension and diabetes, thus suggesting that sociodemographic and clinical factors are associated with dietary intake and should be considered in nutritional interventions.
